# Data Resource Profile: Clinical Practice Research Datalink (CPRD)

**DOI:** 10.1093/ije/dyv098

**Published:** 2015-06-06

**Authors:** Emily Herrett, Arlene M Gallagher, Krishnan Bhaskaran, Harriet Forbes, Rohini Mathur, Tjeerd van Staa, Liam Smeeth

**Affiliations:** ^1^London School of Hygiene & Tropical Medicine, London, UK,; ^2^Clinical Practice Research Datalink, Medicines and Healthcare Products Regulatory Agency, London, UK,; ^3^Utrecht Institute for Pharmaceutical Sciences, Utrecht University, Utrecht, The Netherlands and; ^4^Health eResearch Centre, University of Manchester, Manchester, UK

## Abstract

The Clinical Practice Research Datalink (CPRD) is an ongoing primary care database of anonymised medical records from general practitioners, with coverage of over 11.3 million patients from 674 practices in the UK. With 4.4 million active (alive, currently registered) patients meeting quality criteria, approximately 6.9% of the UK population are included and patients are broadly representative of the UK general population in terms of age, sex and ethnicity. General practitioners are the gatekeepers of primary care and specialist referrals in the UK. The CPRD primary care database is therefore a rich source of health data for research, including data on demographics, symptoms, tests, diagnoses, therapies, health-related behaviours and referrals to secondary care. For over half of patients, linkage with datasets from secondary care, disease-specific cohorts and mortality records enhance the range of data available for research. The CPRD is very widely used internationally for epidemiological research and has been used to produce over 1000 research studies, published in peer-reviewed journals across a broad range of health outcomes. However, researchers must be aware of the complexity of routinely collected electronic health records, including ways to manage variable completeness, misclassification and development of disease definitions for research.

Key Messages

- CPRD data have been extensively used for observational research. For example, the data were used to show that there was no association between MMR vaccine and autism, and to show an association between oral corticosteroid use and increased risk of fractures.
- The CPRD has a large UK dataset bringing together longitudinal primary care medical records from participating practices. Over half of CPRD patients are eligible for linkage to additional datasets, including hospital data, national cancer registration data and national mortality records.
- Quality of some data is driven by the Quality and Outcomes Framework in the UK, and data are also monitored by CPRD internal processes. Analyses described in this paper show that active (alive, currently registered) CPRD patients are representative of the UK population in terms of age and sex.
- CPRD data originate from routine clinical practice, and their use for epidemiological studies typically requires extensive data processing and an understanding of the way the data are originally recorded and stored.

## Data resource basics

### UK primary care data for research

Over 98% of the UK population are registered with a primary care general practitioner (GP)^1^ and under the National Health Service (NHS), visits to the GP are free of charge. The GP is the gatekeeper of care in the UK National Health Service. GPs act as the first point of contact for any non-emergency health-related issues, which may then be managed within primary care and/or referred to secondary care as necessary. Secondary care teams also feed back information to GPs about their patients, including key diagnoses. Patient data are routinely recorded onto computers by practice staff, against a unique patient NHS number. These facets of UK primary care provide good capture of health information in a longitudinal electronic health record.

### The Clinical Practice Research Datalink (CPRD)

The CPRD harnesses general practice data and produces a primary care dataset, which is one of the largest databases of longitudinal medical records from primary care in the world (Table 1). Established in London in 1987, the small Value Added Medical Products (VAMP) dataset grew to become the General Practice Research Database (GPRD) in 1993,^2^^,^^3^ before expanding to become the CPRD in 2012. The CPRD collates routinely collected anonymised electronic health record data from general practices who have agreed at a practice level to provide data on a monthly basis. All patients registered with the participating practices are included in the dataset, unless they have individually requested to opt out of data sharing, by asking their GP to amend their registration details on the system to disable the extraction of their data.

**Table 1.** Key details about the Clinical Practice Research Datalink

Counties participating	UK: England, Wales, Scotland and Nortdern Ireland
Who is included?	Patients registered at general practices that contribute data to CPRD, who have not dissented from secondary use of GP patient-identifiable data
What is recorded?	Demographics, diagnoses, symptoms, signs, prescriptions, referrals, immunisations, behavioural factors, tests
Period of data collection	1987 to present
	Average duration of follow-up 5.1 years
Funding source	CPRD has received funding from the MHRA, Wellcome Trust, Medical Research Council, NIHR Health Technology Assessment programme, Innovative Medicines Initiative, UK Department of Health, Technology Strategy Board, Seventh Framework Programme EU, and various universities, contract research organizations and pharmaceutical companies

### Data linkage

A subset of English practices (currently 75%, representing 58% of all UK CPRD practices) have consented to participate in the CPRD linkage scheme and have provided patient-level information. Patient-level data from consenting practices are linked via a trusted third party (the Health and Social Care Information Centre^4^) to other existing data sources. Established linkages include Hospital Episode Statistics^5^ (hospitalisation data), Office for National Statistics^6^ (mortality data including causes of death), Index of Multiple Deprivation and Townsend scores (deprivation data)and disease registries including the National Cancer Intelligence Network,^7^ and the Myocardial Ischaemia National Audit Project^8^ (details in Supplementary Table 1, available as Supplementary data at *IJE* online). Other linkages are planned (see CPRD website^9^) and researchers can make requests for bespoke linkage for individual studies.

### Uses for observational research and interventional research

Subject to the appropriate data governance and approvals, the CPRD can supply primary care and linked patient data to researchers in the UK and internationally. Through the CPRD, researchers can approach practices and patients to take part in biosample collection studies or trials. The feasibility of this work has been tested: patients from the CPRD have been recruited to a pharmacogenetic study of statin-induced myopathy,^10^^,^^11^ practices have been recruited to cluster randomised trials^12^^,^^13^ and patients have been recruited to pragmatic point-of-care randomised trials.^14^ The electronic health record data can be used alongside the study data to provide a full clinical picture for the recruited patients.

### Ethics

The CPRD has broad National Research Ethics Service Committee (NRES) ethics approval for purely observational research using the primary care data and established data linkages. Other uses of CPRD data may require separate ethical approval. This is likely if there is any specific patient involvement in the study; for example, if the researcher wishes to ask patients to complete a questionnaire for Patient Reported Outcomes, or to conduct an interventional trial among CPRD patients.

### Data governance, practice and patient confidentiality

The CPRD strives to operate within UK and European laws to protect confidentiality. Governance requirements to protect patient confidentiality where patient consent has not been obtained are respected by ensuring that patient identifiers are held separately from the clinical data and that there is separation between researchers with access to identifiable information from the primary study and those using CPRD data.

### Funding sources

The CPRD is a joint venture from the Medicines and Healthcare Regulatory Agency (MHRA) and the National Institute for Health Research (NIHR).The CPRD is owned by the UK Department of Health and operates within the MHRA. The CPRD has received funding for studies from the MHRA, Wellcome Trust, Medical Research Council, NIHR Health Technology Assessment programme, Innovative Medicines Initiative, UK Department of Health, Technology Strategy Board, Seventh Framework Programme EU and various universities, contract research organizations and pharmaceutical companies.

### Data resource area and population coverage

**Figure 1.**
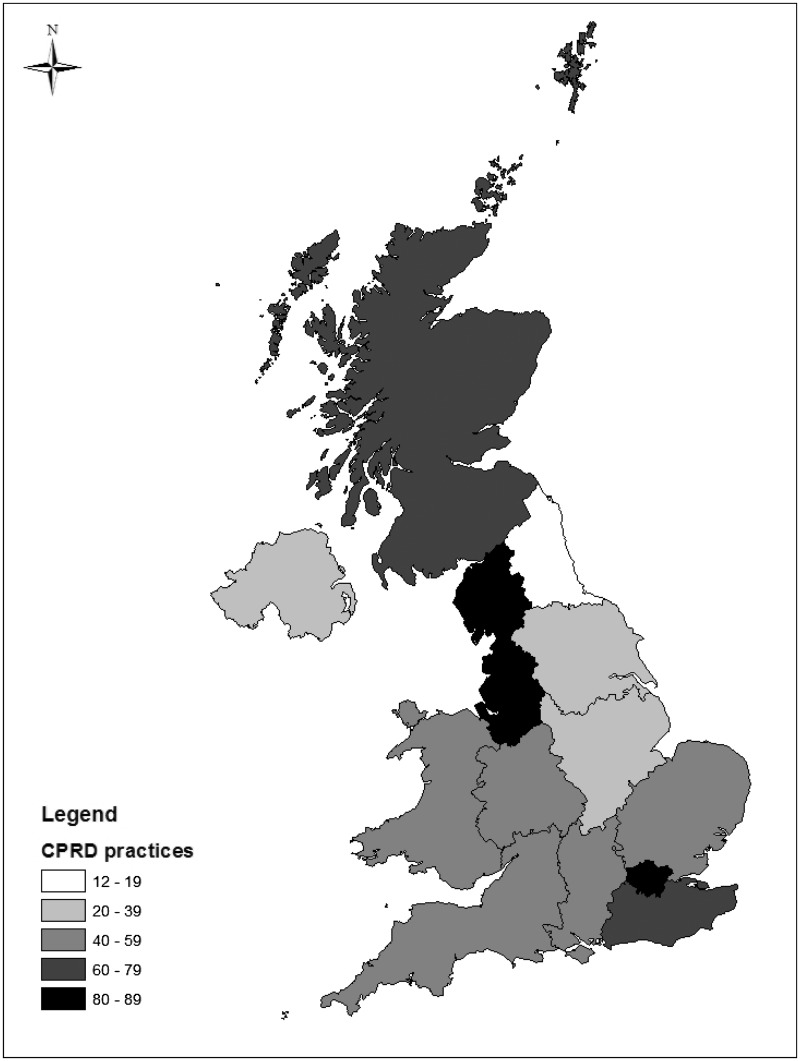
Distribution of 674 CPRD practices by region in England, and in Wales, Scotland and Northern Ireland. Note: practices mapped are those contributing up to standard data to the dataset on 2 July 2013, based on the January 2014 dataset build

Figure 1 describes the population coverage of CPRD primary care data across England, Wales, Scotland and Northern Ireland. At the mid-year date of 2 July 2013, the dataset held information on 11.3 million patients who were deemed acceptable for research based on data quality checks (Appendix 1, available as Supplementary data at *IJE* online, and described below). The population of active patients (alive and currently registered) on 2 July 2013 was 4.4 million, representing 6.9% of the total UK population (based on the UK 2013 mid-year population of 64.1 million). The remaining 6.9 million records represent inactive patients who have died or are no longer registered with a participating practice. Patient numbers by age, sex, deprivation, ethnicity and region are described in Table 2.

**Table 2.** Demographic characteristics of acceptable CPRD patients (January 2014 dataset build), and the subset of those active on 2 July 2013

	All patients	Active
No. patients	11299221	4425016
Men, *n* (%)	5478715 (48.5)	2183161 (49.3)
Women, *n* (%)	5820506 (51.5)	2241855 (50.7)
Age in 2013, *n* (%) (years)		
<18	–	742765 (20.2)
18-64	–	4402926 (61.8)
65+	–	1728514 (18.1)
Region, *n* (%)		
North East	184753 (1.6)	67639 (1.5)
North West	1257846 (11.1)	523356 (11.8)
Yorkshire & The Humber	441933 (3.9)	48480 (1.1)
East Midlands	446799 (4)	29954 (0.7)
West Midlands	943011 (8.4)	394115 (8.9)
East of England	1117235 (9.9)	306538 (6.9)
South West	943295 (8.4)	377821 (8.5)
South Central	1236351 (10.9)	544979 (12.3)
London	1532066 (13.6)	600824 (13.6)
South East Coast	1130468 (10)	474593 (10.7)
Northern Ireland	275640 (2.4)	153576 (3.5)
Scotland	960121 (8.5)	499969 (11.3)
Wales	829703 (7.3)	403172 (9.1)
Duration of follow-up (median years, IQR)^a^	5.1 (1.8-11.1)	9.4 (3.4-13.9)

Active patients are alive and currently registered on 2 July 2013.

^a^Includes only up to standard follow-up.

### Frequency of data collection

Data collection happens as part of normal clinical care of patients in participating practices on a daily basis. The frequency of data recording is determined by patient need and varies by age, sex and underlying morbidity. Patients are included in the primary care dataset from their first until their last contact with the participating practice. Data are collected by practices and usually uploaded to the CPRD secure servers on a monthly basis. The date of last data collection corresponds to the date of the last data upload from each practice. Monthly builds of the primary care dataset are generated and made available for researchers to use.

## Measures

### Practice and patient data

The database structure broadly separates information into clinical, referral, immunisation, test and therapy data (see Supplementary Table 1, available as Supplementary data at *IJE* online). Data are recorded against practice and patient pseudo-identifiers. At the practice level, geographical region is recorded by the CPRD as one of 10 regions in England, with Wales, Scotland and Northern Ireland as separate regions (Figure 1); a practice-level deprivation score is also calculated based on practice lower super output area.

All general practice encounters are recorded electronically and practitioners are encouraged to make these records available for research. Data are collected on demographic information, prescription details, clinical events (symptoms, diagnoses), preventive care provided, tests, immunisations, specialist referrals, hospital admissions and their major outcomes, and details relating to death (details are shown in Supplementary Table 2, available as Supplementary data at *IJE* online).

**Figure 2.**
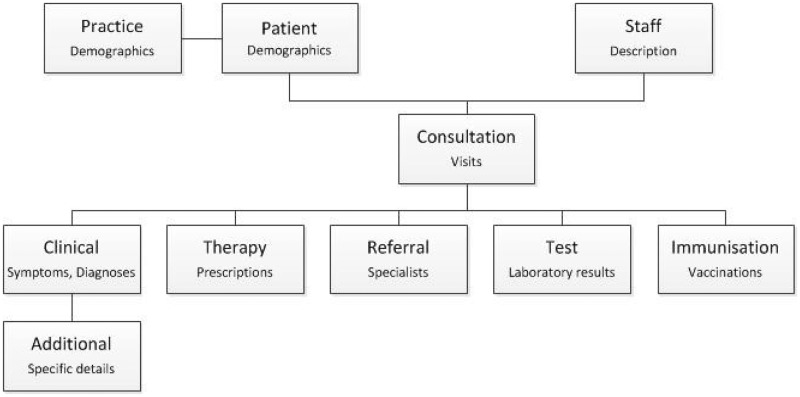
Example of dataset structure. Note: patients consult with practice staff, where clinical, therapy, referral, test and immunisation information is coded in the medical record.

All entries to a patient record are considered as ‘consultations’, not all of which will involve a face-to-face encounter. Within a consultation multiple ‘events’ may be recorded, each with an associated date (Figure 2).

Data are largely recorded by general practice staff using version 2 Read codes, a hierarchical clinical classification system containing over 96, 000 codes.^15^ For example, during a consultation, a GP, nurse, other healthcare professional, practice manager or administrator may enter a number of Read codes to describe a patient’s condition (e.g. lifestyle measures such as smoking status, symptoms, past medical history, diagnoses, tests performed such as blood pressure measurement, and therapies offered). Numerical data on additional clinical measures (e.g. height, weight, blood pressure, alcohol intake) can also be recorded during consultations. Prescriptions issued by the GP are automatically recorded with a product name and British National Formulary code, alongside the dosage instructions and quantity. Results of laboratory tests ordered by the GP are commonly added to the patient record via electronic links to laboratories. Data fed back to the GP from other sources may also be entered into the patient record by practice staff; this might include information from secondary care such as key diagnoses, discharge data from hospitals, or follow-up information from specialist clinics.

The GP is also able to make additional uncoded notes and observations about patients as free text. This often contains identifiable information and is not part of the standard database available to researchers.

## Data resource use

Data from the CPRD (or formerly the GPRD or VAMP) have been used in the UK and internationally^16^ to produce close to 2000 research reports, with over 1000 published in peer-reviewed journals, across all major therapeutic areas. A bibliography is maintained by the CPRD and is available online.^17^ These publications cover a range of health-related research topics including pharmacoepidemiology, comparative effectiveness research, health services research, assessments of temporal trends in disease incidence, health economics, prognosis research, classical risk factor epidemiology and more recently randomised controlled trials.^12^^,^^18^ Publications to date include studies showing the absence of an association between measles, mumps and rubella (MMR) vaccine and autism,^19^ cardiovascular risk after acute infection,^20^ the lower risk of dementia associated with statin use,^21^ the risk of myocardial infarction in patients with psoriasis,^22^ the use of oral corticosteroids and fracture risk^23^ and the association between body mass index and cancer.^24^

## Strengths and weaknesses

### Strengths

The strengths of the CPRD data as a research resource lie in the breadth of coverage, size, long-term follow-up, representativeness and data quality.

#### Breadth of data

The CPRD primary care dataset is one of few large, ongoing databases that include data on morbidity and lifestyle variables and with a linkage to secondary care and mortality data.

#### Size and long- term follow-up

A key strength of this database is its size; the CPRD holds data from 674 practices and includes over 79 million person-years of follow-up (on 2 July 2013, January 2014 dataset). This allows epidemiological associations to be investigated in more detail and estimated with a higher level of statistical precision than is possible with smaller data sources, which is of particular importance for the study of rare exposures and diseases.^25^^,^^26^ For individual patients, there is a median prospective follow-up of 9.4 years for active patients [interquartile range (IQR) 3.4–13.9] and 5.1 years (IQR 1.8–11.1 years) (Table 2) overall, enabling research into diseases with long latency and the study of long-term outcomes.^27–29^

#### Representativeness

**Figure 3.**
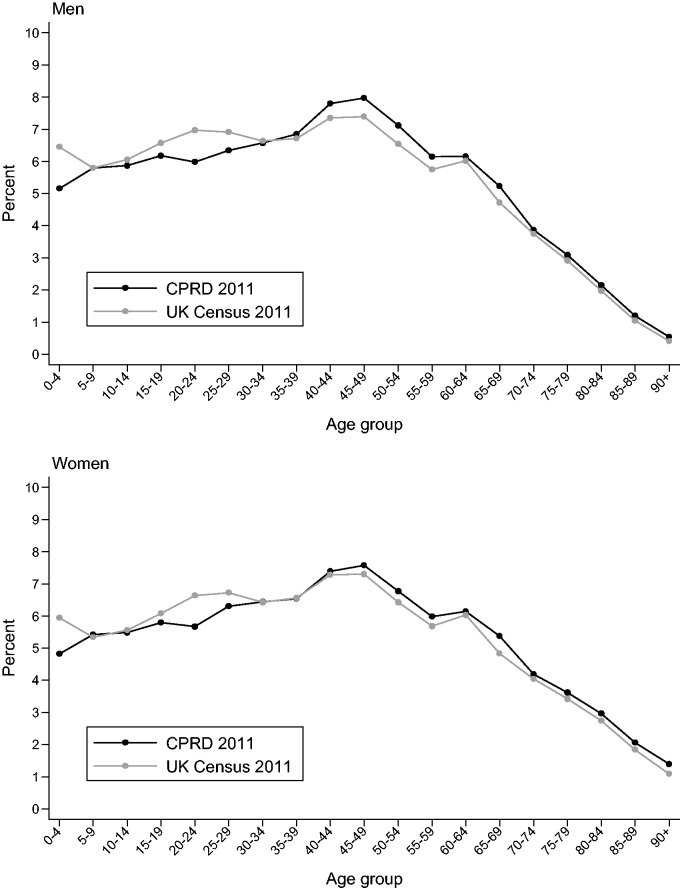
Age distribution of the CPRD primary care data on 27 March 2011 compared with UK Census data 2011, in men (top panel) and women (lower panel). These data are based on a one-million patient sample of CPRD. All patients are acceptable.

When compared with the UK census in 2011,^30^ CPRD patients are broadly representative of the UK population in terms of age and sex (Figure 3). Patients are also comparable to the UK census in terms of ethnicity,^31^ and comparable to the Health Survey for England for body mass index distribution in most patient subgroups.^32^ However, the CPRD may not be representative of all practices in the UK based on geography and size.^33^

#### Data quality

**Figure 4.**
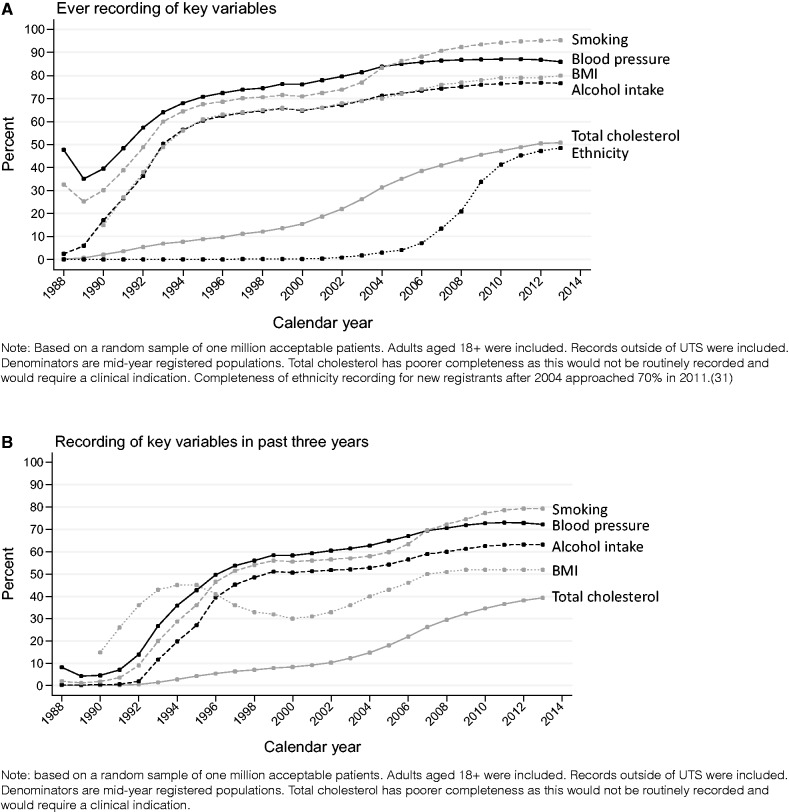
Recording of key lifestyle and demographic variables by calendar year (A: ever recorded in patient follow-up; B: recorded in the past 3 years of patient follow-up). These data are based on a one-million patient sample of primary care data from the CPRD. All patients are acceptable.

Aspects of data quality in English general practice are enhanced by the Quality and Outcomes Framework,^34^ an incentive payment programme for GPs, which encourages recording of key data items (for example smoking status and the delivery of services to key patient groups). The Quality and Outcomes Framework was introduced in 2004, and completeness in recording of many variables showed subsequent improvement (Figure 4, and Supplementary Figure 1, available as Supplementary data at *IJE* online).

Validation of the CPRD has shown high positive predictive value of some diagnoses and, where evaluated, comparisons of incidence with other UK data sources are also broadly similar.^35–38^ However, reporting of validation studies was often too poor to permit a clear interpretation, and the majority of studies focused on positive predictive value rather than sensitivity or specificity.^39^

The quality of primary care data is variable because data are entered by GPs during routine consultations, not for the purpose of research. Researchers must therefore undertake comprehensive data quality checks before undertaking a study. The CPRD provides two sets of data quality criteria: acceptability for patients and up to standard (UTS) time for practices. These criteria do not ensure data quality, but the CPRD recommends that these measures are used as a first step to selecting research-quality patients and periods of quality data recording. The acceptable patient metric is based on registration status, recording of events in the patient record, and valid age and gender. The UTS date is a practice-based quality metric based on the continuity of recording and the number of recorded deaths. The UTS date is calculated for each participating practice, corresponding to the latest date at which practices meet these minimum quality criteria (Appendix 1, available as Supplementary data at *IJE* online). The figures given in this paper reflect data for patients labelled as acceptable and who have at least 1 day of follow-up that is ‘up to standard’. Research into data quality has shown that, despite these criteria, there were large variations in inter-practice recording of data.^40^

## 

### Weaknesses

#### Missing data

The variability in completeness of data across patients and across time requires careful consideration; restriction to those with complete data may result in biased analyses, and imputation may not be a straightforward approach because the patterns of missingness are complex. For example, body mass index may be recorded more frequently in patients with a health issue, and blood pressure more frequently in women of reproductive age and those with existing cardiovascular disease. Complex algorithms are often required to deal with missingness, to resolve discrepancies in measures between consultations and to decide whether historical measurements, for example of body mass index, blood pressure or smoking status, are still appropriate to a patient’s disease risk much later in follow-up.^32^

An additional complexity of primary care data is that the absence of a Read code for disease must be interpreted as an absence of the disease itself, so whereas positive predictive value tends to be high,^39^ sensitivity may be lower. This potential misclassification arises partly due to patients failing to present to the GP with disease, and also from variations between GPs in coding diagnoses in the patient electronic record; if GPs enter information as free text, researchers will miss valuable information. The extent of misclassification may vary between diseases.^39^

#### Definitions

There are not generally standardised definitions for diagnoses and other details, so Read code lists and algorithms need to be developed for each study to identify exposures and outcomes of interest. This may lead to inconsistent definitions (and therefore results) between studies using the same data.

#### Information from secondary care

General practices receive information about patient contacts with secondary care but this information must be manually entered into the patient record. Therefore, details about hospital admissions (dates, diagnoses, tests performed, length of stay) may be incomplete.

#### Data not captured

Some aspects of health may be recorded very infrequently or not at all, for example level of social support, number of people in a household, over-the-counter medication use, prescriptions in secondary care, prescriptions filled, and adherence to treatments. There are also certain patient groups that are missing from primary care records, such as prisoners, private patients, some residential homes and the homeless.

## Data Resource access

Access to patient level data is provided by the CPRD for health research purposes and is dependent on approval of a study protocol by the MHRA Independent Scientific Advisory Committee (ISAC).

Researchers intending to use the data should be aware that the CPRD data files contain millions of rows of data, requiring extensive data management and an in-depth understanding of the way the data are input and stored.

The CPRD provide data dictionaries and coding dictionaries to researchers, and guidance on creating code lists is available to help identify codes of interest.^41^ Read code repositories for electronic health record research are also now available.^42^^,^^43^

Details about ISAC applications and data costs are available on the CPRD website, and any other queries can be directed to the CPRD Knowledge Centre [kc@cprd.com].^9^

## Supplementary Data

Supplementary data are available at *IJE* online.

## Funding

LS is supported by a Wellcome Trust Senior Research Fellowship in Clinical Science grant number 098504/Z/12/Z.

***Conflict of interest:**** AG is employed by the CPRD and TvS is an ex-employee of the CPRD. No other conflicts declared.*

## Supplementary Material

Supplementary Data
