# Supplementary material for: Data Resource Profile: Clinical Practice Research Datalink (CPRD)
Source: Int J Epidemiol. 2015 Jun 6;44(3):827–36. doi: 10.1093/ije/dyv098 (PMC4521131; doi:10.1093/ije/dyv098)
Supplement: Supplementary Data [file supp_44_3_827__index.html]

Data Resource Profile: Clinical Practice Research Datalink (CPRD) — Supplementary Data 

# Data Resource Profile: Clinical Practice Research Datalink (CPRD)

## Supplementary Data

files

- Supplementary Data - zip file
