# Supplementary material for: Data Resource Profile: Clinical Practice Research Datalink (CPRD)
Source: Int J Epidemiol. 2015 Jun 6;44(3):827–36. doi: 10.1093/ije/dyv098 (PMC4521131; doi:10.1093/ije/dyv098)
Supplement: Supplementary Data [file supp_dyv098_suppl_data.zip › ije-2015-02-0212-File001.docx]

**Supplementary material**

**Supplementary Table 1.** Data sources linked to CPRD primary care records. These data are available for 75% of English practices (58% of all practices) in CPRD.

| Linkage | HES inpatient | HES outpatient | MINAP | ONS | National Cancer Data Repository (NCDR) | Deprivation |
| --- | --- | --- | --- | --- | --- | --- |
| Type of resource | Hospitalisation data for inpatients | Outpatient data | Acute coronary syndrome disease registry | Date and cause of death register | Cancer registry | Socioeconomic status |
| Who is included? | Patients with hospitalisations for any cause | Patients presenting in outpatients | Patients hospitalised with acute coronary syndrome in NHS hospitals | People who die in England and Wales | Tumour level records submitted to Office of National Statistics (ONS) by the England Cancer Registries | Lower super output area levels |
| Geographic regions covered by linkage | England | England | England and Wales | England and Wales | England | England, Wales, Scotland and Northern Ireland |
| Period of linkage | 1997 onwards | 2003 onwards | 2003 onwards | Cause of death recorded since 1841, but since 2001 using ICD-10 codes, | 1990 onwards | Calculated at different times for each country |
| Examples of data available in linked dataset | Diagnoses, procedures | Diagnoses | Details regarding ACS diagnosis and management | Date and cause of death, including underlying and secondary causes. | Cancer type and date of diagnosis | Townsend Scores (material deprivation), Index of Multiple Deprivation (IMD) |
| How are data coded | ICD-10 and OPCS-4 | ICD-10 | In 120 fields with multiple response categories as defined by the MINAP steering group. | ICD-10 | ICD-10 | Townsend Scores, IMD – in quintiles |
| Permissions required | ISAC | ISAC | ISAC, MINAP academic group | ISAC | ISAC, PHE | ISAC |
| Additional cost for data? | Yes | Yes | Yes | No | Yes | No |
| ICD-10: International Classification of Disease, version 10; OPCS-4: Office of Population, Censuses and Surveys Classification of Surgical operations and procedures, version 4 codes | | | | | | |

**Supplementary Table 2** Data files supplied by the Clinical Practice Research Datalink

| **File type** | **What it holds** | **Example of contents** |
| --- | --- | --- |
| Patient | Demographic and registration status of patients | Patient identifier, month and year of birth, registration status, death date, transfer out date |
|  |  |  |
| Practice | Practice administrative data | Practice identifier, geographical region, date practice became 'Up to standard', last data collection date |
|  |  |  |
| Staff | Information about the staff members entering data | Staff identifier, gender, role |
|  |  |  |
| Consultation | Administrative information about the consultation | Date of clinical event, date of data entry, type of consultation, staff identifier and duration of consultation |
|  |  |  |
| Clinical | Clinical data regarding medical history | Date of clinical event, date of data entry, the CPRD medical code for the chosen Read code, additional details identifier*, entity type |
|  |  |  |
| Additional Clinical Details (ACD) | Specific data about a clinical event | Type of information held, called an 'entity', specific clinical details relating to that entity |
|  |  |  |
| Referral | Details on referrals to secondary care or specialists | The CPRD medical code for the chosen Read code, method of referral, referral specialty, urgency of referral |
|  |  |  |
| Immunisation | Data associated with immunisations | Reason for immunisation, type, stage, status and the compound used |
|  |  |  |
| Test | Test results | Type of test, result, normal range of result, unit of measure |
|  |  |  |
| Therapy | Information about therapies including medications and appliances | The CPRD product code for the medication, British National Formulary code, quantity of product, dose, pack size, number of days prescribed |
|  | | |
| *Allows a link to be made between a Read code in the 'clinical file' to additional details held in the 'additional clinical details' file. | | |

**Supplementary Figure 1a.**

**Supplementary Figure 1b.**

**Supplementary Figure 1c.**

**Supplementary Figure 1d.**
