# Supplementary material for: Data Resource Profile: Clinical Practice Research Datalink (CPRD)
Source: Int J Epidemiol. 2015 Jun 6;44(3):827–36. doi: 10.1093/ije/dyv098 (PMC4521131; doi:10.1093/ije/dyv098)
Supplement: Supplementary Data [file supp_dyv098_suppl_data.zip › ije-2015-02-0212-File003.doc]

**Glossary of terms/Data definitions**

**Acceptable Patients**

Patients are labelled as ‘acceptable’ for use in research by a process that identifies and excludes patients with non-continuous follow up or patients with poor data recording that raises suspicion as to the validity of the that patients record. Patient data is checked, for the following issues:

- An empty or invalid first registration date
- An empty or invalid current registration date
- Absence of a record for a year of birth
- A first registration date prior to their birth year
- A current registration date prior to their birth year
- A transferred out reason with no transferred out date
- A transferred out date with no transferred out reason
- A transferred out date prior to their first registration date
- A transferred out date prior to their current registration date
- A current registration date prior to their first registration date
- A gender other than Female/Male/Indeterminate
- An age of greater than 115 at end of follow up
- Recorded health care episodes in years prior to birth year
- All recorded health care episodes have empty or invalid event dates
- Registration status of temporary patients

If any of these conditions are true then the patient is labelled unacceptable, and is not recommended for use in research.

**UTS date**

The overall quality of data in practices is mediated by use of an ‘up to standard’ (UTS) date, which is deemed as the date at which data in the practice is considered to have continuous high quality data fit for use in research. This is mediated by an analysis on the total data in the practice, which is refreshed every time a new collection for a practice is processed into the database. It is based on two central concepts: assurance of continuity in data recording (gap analysis), and avoidance of use of data for which transferred out and dead patients have been removed (death recording).

Gap Analysis

To detect whether there is any meaningful gaps in the data it is necessary to look in more detail at single day gaps as well as longer gaps. A single day alone may reflect a situation where nothing was recorded that day at the practice, i.e. the practice was not open, such as on a bank holiday. A longer gap may reflect a situation where the practice did not offer a service and patients may have been treated elsewhere. If a meaningful gap is found, the earliest date after which there is no significant gap is identified.

Death Recording

It is expected that a standard number of deaths will be recorded at a practice over time. Assessment of gaps in death recording is performed taking the size of the practice into account. A safety margin is built in to account for both geographical and seasonal variation in death rates. If a meaningful gap is found, the earliest date after which there is no significant gap is identified.

The UTS date is set to the latest of these dates for each practice. The CPRD recommend that analyses are performed on data following the practice UTS date.
